# The Multifaced Actions of Curcumin in Pregnancy Outcome

**DOI:** 10.3390/antiox10010126

**Published:** 2021-01-17

**Authors:** Giovanni Tossetta, Sonia Fantone, Stefano Raffaele Giannubilo, Daniela Marzioni

**Affiliations:** 1 Department of Experimental and Clinical Medicine, Università Politecnica delle Marche, 60126 Ancona, Italy; g.tossetta@pm.univpm.it (G.T.); s.fantone@pm.univpm.it (S.F.); 2 Clinic of Obstetrics and Gynaecology, Department of Clinical Sciences, Università Politecnica delle Marche, Salesi Hospital, Azienda Ospedaliero Universitaria, 60126 Ancona, Italy; s.giannubilo@univpm.it

**Keywords:** pregnancy, curcumin, preeclampsia, Fetal Growth Restriction (FGR), Gestational Diabetes Mellitus (GDM), preterm birth (PTB)

## Abstract

Curcumin, also known as diferuloylmethane, is the main polyphenolic substance present in the rhizomes of *Curcuma longa L*. This plant showed many beneficial effects and has been used since ancient times for both food and pharmaceutical purposes. Due to its pleiotropic functions, curcumin consumption in the human diet has become very common thanks also to the fact that this natural compound is considered quite safe as it does not have serious side effects. Its functions as an anti-inflammatory, anti-oxidant, neuroprotective, immunomodulatory, anti-toxicant, anti-apoptotic, and anti-diabetic compound are already known and widely demonstrated. There are numerous studies concerning its effects on various human pathologies including cancer, diabetes and arthritis while the studies on curcumin during pregnancy have been performed only in animal models. Data concerning the role of curcumin as anti-inflammatory compound suggest a possible use of curcumin in managing pregnancy complications such as Preeclampsia (PE), Gestational Diabetes Mellitus (GDM), Fetal Growth Restriction (FGR), PreTerm Birth (PTB), and exposure to toxic agents and pathogens. The aim of this review is to present data to support the possible use of curcumin in clinical trials on human gestation complications.

## 1. Introduction

**Figure 1:**
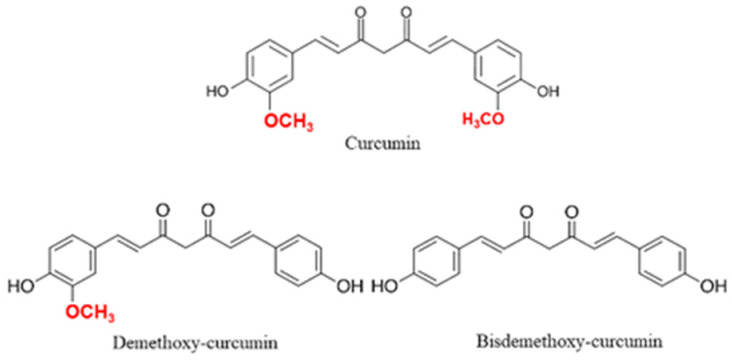
Curcuminoids chemical structures.

Curcumin (1,7-bis(4-hydroxy-3-methoxyphenyl)-1,6-heptadiene-3,5-dione), also known as diferuloylmethane, is a polyphenolic yellow substance coming from the rhizomes, the most commonly used plant part, of *Curcuma longa Linn* (*Zingiberaceae* family) [1,2]. Curcumin structure is similar to other bioactive non-volatile curcuminoids such as dimethoxy-curcumin and bisdemethoxy-curcumin, differing only for the number of methoxy groups on their aromatic rings (Figure 1) [3]. This plant has been used since ancient times for both food and pharmaceutical purposes, showing a variety of beneficial effects in the organism reducing glycemia [4], hyperlipidemia [5], insulin resistance [6] and steatosis in non-alcoholic fatty liver disease (NAFLD) [7]. Moreover, due to the cytotoxic effects of curcumin on tumor cells, this compound showed good effects also as an anticancer agent inhibiting tumor proliferation and inducing apoptosis in many cancer types including breast [8,9], colon [10], lung [11] and gastric cancer [12]. In addition, curcumin showed anti-metastatic [13], radioprotective [14,15] and chemosensitizer effects reducing the adverse effects of chemotherapeutic drugs [16,17] and leading to the use of nano-formulations of curcumin for the treatment of cancer [18]. Curcumin is generally recognized as a safe substance and its use in humans did not show any toxic effects at the dose of 6 g/day orally for 4–7 weeks [2].

Pregnancy requires several steps tightly regulated where the placenta is an essential organ undergoing continuous remodeling during fetal development [19,20,21]. The placenta has a multifaceted role in the normal development of humans and mammals in general, playing essential functions during pregnancy [22,23]. Its importance is evident when placental development is impaired, leading pathological conditions such as Preeclampsia (PE) [24,25,26,27], Fetal Growth Restriction (FGR) [28,29,30], PreTerm Birth (PTB) [31,32,33], Gestational Diabetes Mellitus (GDM) [34,35], pregnancy loss [36,37,38] and more serious pathologies such as e.g., choriocarcinoma [39]. In addition, normal placental development could be impaired by exposure to toxic agents, virus and bacteria [40,41,42,43] as in chorioamnionitis.

Curcumin has been extensively studied in various fields, showing a wide range of action, including

Antioxidant, anti-inflammatory, anti-toxicant, anti-apoptotic, anti-diabetic and immunomodulatory actions, demonstrated by in vitro studies and animal models, suggest the use of this compound as a therapeutic agent in counteracting several pregnancy complications [44,45,46,47,48,49]. Inflammation, oxidative stress, Reactive Oxygen Species (ROS) generation and apoptosis are common conditions usually found in almost all of the pathological placental conditions mentioned above; for this reason, curcumin could play a key role in improving pregnancy outcome in these complications.

The purpose of this review is to provide an overview of the potential health benefits and possible adverse effects of curcumin, evaluating a possible role of this natural compound in ameliorating or preventing pregnancy complications and improving pregnancy outcome.

## 2. Curcumin Effects on Fertilization and Fetal Development

Oocyte fertilization and fetal development are complex processes tightly regulated and conditioned by the microenvironment [20]. For this reason, chemical injury may impair these processes leading to developmental problems [50]. Despite the fact that the beneficial effects of curcumin have been highlighted in many in vitro and in vivo studies and that it has been proven safe in humans, there are still conflicting studies regarding its effect on oocyte maturation, fertilization and development of the blastocyst. Chen and colleagues examined the cytotoxic effects of curcumin by treating mouse blastocysts with different concentrations of curcumin. Interestingly, they found a significantly increased apoptosis and decreased total cell number in blastocysts treated with 24 µM curcumin for 24 h. Moreover, they found a decreased implantation rate as well as decreased fetal weight in the embryos when blastocysts treated with curcumin were transferred in mouse uterus, demonstrating a cytotoxic effect of curcumin on blastocysts by increasing mitochondria-dependent apoptotic signaling processes trough ROS generation, impairing embryonic development [51]. These data were partly supported by Huang and colleagues which found that exposure to 24 μM of curcumin at the blastocyst stage was lethal for all embryos [52]. Further studies of Chen and colleagues showed a significant reduction in the rate of oocyte maturation, fertilization, and in vitro embryonic development in oocytes treated with 20 µM curcumin for 24 h. Interestingly, injury effects of curcumin were prevented when caspase-3-specific inhibitor pre-treatment was present, suggesting that embryo impairment by curcumin is mainly due to the activation of a caspase-dependent apoptotic process [53]. Other studies showed no adverse effects on reproductive capacity in mice and rats fed with curcumin [54,55]. Interestingly, in vitro study of spermatozoa coming from patients affected by asthenozoospermia showed that 100-nM curcumin treatment increased spermatozoa motility and reduced ROS formation and malondialdehyde (MDA) production increasing Nrf2 expression compared to untreated spermatozoa. However, higher concentrations (1 mM and 1 M) reduced spermatozoa motility [56]. These dose-dependent effects of curcumin on human spermatozoa motility were further proven in another study testing high concentrations of curcumin (31,25 µM–500 µM) on human normal spermatozoa showing a reduced spermatozoa motility starting at a concentration of 62.5 µM. In addition, a reduced capacitation/acrosome reaction was detected in all concentrations tested [57].

These studies provide contrasting data on curcumin intake in early pregnancy in animal models, suggesting a prudent use of curcumin during the early stage of gestation. Moreover, high curcumin intake may affect spermatozoa motility, impairing oocyte fertilization.

## 3. Curcumin as Protector of Cytotoxic and Teratogenic Agents

Placental growth and fetal development are complex processes tightly regulated during pregnancy and are affected by the microenvironment changes due to the exposure to chemical compounds whose effects can be substantially attenuated by the use of antioxidant substances

Retinoic Acid (RA) is an oxidative metabolite of vitamin A associated to a group of birth defects called Retinoic Acid Embryopathy (RAE) and consisting of thymic, cardiac, and central nervous system malformations due to a prenatal exposure to RA [58,59,60]. Interestingly, Barandeh and colleagues treated pregnant mice with 10 mg/kg curcumin in the presence or absence of 60 mg/kg RA and highlighted a significant increase in the lengths of crown rump (CR) and embryo weights in the group treated with a combination of curcumin and RA, suggesting a key role of curcumin in antagonizing the side effects of RA [44].

PolyChlorinated Biphenyls (PCBs) belong to a broad family of organic compounds exposure to which leads to health problems including cancer [61,62], endometriosis [63] and neurologic problems [64,65]. PCBs can pass through the placenta reaching the fetus but they can also be released in the human milk [66] and reach the newborn having significant toxicologic effects by interrupting the neurodevelopmental processes [67]. Interestingly, the cerebellar and cerebral cortex tissue homogenates of rats treated with curcumin and Aroclor1 254 (a PCBs mixture) showed lower levels of 8-Hydroxy-2′-deoxyguanosine (8-(OH)DG) (a marker of DNA damage) compared to rats treated with Aroclor 1254 alone [68]. These results suggested neuro-protective effects of curcumin against DNA damage due to PCBs exposure during the prenatal period.

Lead (Pb) is an environmental pollutant [69] involved in important pediatric health problems as it can cross the placental and blood–brain barrier leading to neurotoxic effects [70,71]. These effects may be due to enzymatic, genetic and oxidative damages caused by Pb exposition [72]. Interestingly, Benammi at colleagues studied the possible role of curcumin as neuro-protective compound in rats exposed to Pb during gestation and showed that curcumin was able to restore the neuronal and locomotor behavior alterations induced by lead intoxication [73], suggesting curcumin as potent neuro-protective compound against lead-induced neurotoxicity.

Intrauterine alcohol exposure, due to pregnant women who drink alcohol during pregnancy, is a well-established cause of Fetal Alcohol Syndrome (FAS), a group of disorders characterized by facial dysmorphism, cardiac defects, fetal growth restriction and neurodevelopmental delays [74]. Although the causes of some of these disorders are still unknown, heart defects appear to be associated with impaired acetylation of histone H3K14 leading to the overexpression of specific cardiac genes such as the basic helix–loop–helix transcription factors: DHAND and EHAND. Interestingly, it has been shown that curcumin administration to pregnant C57BL/6 mice exposed to alcohol was able to inhibit Histone acetyltransferase (HAT) activity reducing H3K14 acetylation and leading to a reduced expression of DHAND and EHAND in the fetal heart [75]. In a further study, the same research group proved that alcohol exposure during pregnancy decreased the acetylation of histone H3K9 near the promoter region of bcl-2 and increased the acetylation of histone H3K9 near the promoter region of caspase-3 and caspase-8, leading to increased apoptotic levels in the embryonic hearts. However, combined intake of curcumin during pregnancy could reverse this condition, correcting the high level of histone H3K9 acetylation induced by alcohol exposure [76]. These results proved a key role of curcumin in preventing fetal heart defects in mice exposed to alcohol during pregnancy.

Arsenic (As) is a ubiquitous metalloid element present in sediments and soils. However, from these deposits, it can be easily dissolved to the nearby aquifers. Therefore, drinking water contaminated by arsenic can expose humans and animals to its toxic effects [77]. To date, there is convincing evidence that links arsenic exposure during pregnancy to spontaneous abortion, stillbirth, neonatal death, post neonatal death, preterm delivery and low birth weight [78]. Although the effects of arsenic exposure during pregnancy are well known, the mechanisms by which arsenic acts are still unknown. When pregnant mice were exposed to drinking water containing arsenic there was an increased apoptosis of the multipotent adult stem cell (EpASCs isolated from epidermis of neonate mouse skin) and increased levels of Nrf2, NFkB, IkB, TNF-α proteins. Interestingly, the combined intake of curcumin during pregnancy was able to prevent the disruption of homeostasis and associated biochemical changes [79], indicating curcumin as a possible compound capable of counteracting the cytotoxic effects of arsenic exposure during pregnancy.

Celecoxib, a selective cyclooxygenase-2 (COX-2) inhibitor, is a pharmacologic compound used to treat pregnant women suffering from pelvic pain and inflammatory diseases [80,81]. However, using celecoxib during pregnancy could impair fetal brain development [82] since COX-2 plays a pivotal role in neural progenitor cell differentiation and proliferation [83]. In a recent study, Rong and colleagues showed that intrauterine exposure to celecoxib inhibited the Wnt/β-catenin pathway altering the proliferation of neuronal progenitor cells and impairing newborn neurons in fetal frontal cortex. Interestingly, this effect could be attenuated when pregnant mice were exposed to curcumin, activating the Wnt/β-Catenin pathway [84]. This study clearly proved a neurotoxic effect of celecoxib in pregnancy and suggest a therapeutic role of curcumin in treating celecoxib-induced neurotoxicity in pregnancy.

Mercury (Hg) is a nonessential metal in biological processes but the exposure to this metal is highly toxic for humans and animals, damaging many organs and tissues [85]. Moreover, chronic exposure to mercury during pregnancy leads to impaired sensorimotor function and decreased weight gain [86]. Abu-Taweel reported the effects of mercuric chloride (HgCl_2_) in pregnant mice exposed to this compound and reported a decreased body weight, memory and learning deficits, anxiety behavior, and decreased level of dopamine (DA), serotonin (5-HT) and acetylcholinesterase (AChE) in forebrain of the pups. However, administration of curcumin improved biochemical and behavioral disorders in HgCl_2_ treated animals [87], suggesting that curcumin can be used to improve health conditions in case of exposure to mercury and other heavy metals. Moreover, the same research group showed that low concentrations of curcumin (5–10 µM) were able to inhibit methylglyoxal-induced ROS generation and subsequent apoptosis in mouse embryonic stem cells ESC-B5 and blastocysts isolated from pregnant mice, suggesting a protective role of curcumin on blastocyst development [46].

All these studies suggested a potential role of curcumin in counteracting the effects of cytotoxic and teratogenic agents on fetal development ameliorating pregnancy outcome in animal models. No data are available on human gestation.

## 4. Curcumin as Potential Treatment of Viral and Bacterial Infections

Congenital infections are usually caused by viruses or bacteria that infect the mother during pregnancy and are transmitted to the fetus during pregnancy. Congenital infections may have potentially severe pregnancy complications such as PTB, FGR or miscarriage [42,43]. For this reason, treating these infections becomes fundamental for a successful pregnancy outcome. Interestingly, Mounce and colleagues proved inhibiting effects of curcumin in Zika virus (ZIKV) infection by blocking the early stage of infection, in particular by inhibiting virus binding to the cell surface [88]. This effect of curcumin has been reported on several epidemic human strains of ZIKV [89]. In addition, it has been proven that curcumin could inhibit viral infection also by other mechanisms. In fact, Lv and colleagues showed that curcumin could inhibit Cytomegalovirus (CMV) activity by downregulating heat shock protein 90 (Hsp90) improving the survival rate of the host cells [90]. Moreover, it has been shown that curcumin may impair herpes simplex virus type 1 (HSV-1) infection by inhibiting the viral trans activator protein VP16 and blocking recruitment of RNA polymerase II to the immediate early (IE) gene promoters of HSV-1 [91]. In addition, it has been proven that curcumin was able to inhibit human immunodeficiency viruses (HIV) infection by promoting the trans-activator of transcription (tat) degradation [92] or blocking HIV integrase [93].

The beneficial effects of curcumin have also been seen in preventing and combating infection of bacteria commonly involved in pregnancy. For example, it has been shown that curcumin was able to influence listeriolysin O (LLO) oligomerization, counteracting *Listeria monocytogenes* infection in animal models [94]. Moreover, curcumin may protect from *Streptococcus agalactiae* infection by stimulating immune system response although the mechanism is still unknown [95].

In addition, curcumin has been suggested as anti-microbial compound in treating *Neisseria gonorrhoeae* infection. In fact, curcumin was able to inhibit the release of pro-inflammatory cytokines and attenuate adhesion of the bacterium to the host cells in late infection [96].

The protective effects of curcumin in bacterial infections may primarily be due to its ability in modulating host immune response to bacterial virulence factors such as LipoPolySaccharide (LPS). In fact, by using animal models, it has been proven that curcumin was able to reverse the release of several pro-inflammatory cytokine after administration of LPS to pregnant mice [97].

At present no data are available on curcumin in human pregnancy infections but the in vitro and animal model studies mentioned above suggest a potential role of curcumin in inhibiting, treating and preventing viral and bacterial infections.

## 5. Curcumin in Gestational Diabetes Mellitus (GDM)

Worldwide, impaired maternal glucose regulation occurs in about 15% of pregnancies [98]. Gestational Diabetes Mellitus (GDM), defined as glucose intolerance of variable degree with onset or first recognition during pregnancy, is generally diagnosed at 24–28 weeks of gestation [34,99] and is a major cause of miscarriage, preeclampsia, congenital malformations, macrosomia and preterm labor. Moreover, GDM may lead to diabetes, obesity, and metabolic dysfunction in both mother and child [98,100]. For these reasons, early treatment, or better yet, prevention of GDM becomes essential for good pregnancy outcome.

Many studies in vivo and in vitro elucidated the role of curcumin in reducing inflammation, oxidative stress and insulin resistance, suggesting a possible role of this natural compound in alleviating diabetes and its complications. The hyperglycaemic condition in GDM impairs intracellular molecular activities and organelle functions such as mitochondria, endoplasmic reticulum (ER) leading to protein misfolding, increased production of reactive oxygen species (ROS) and inhibiting antioxidant enzymes such as superoxide dismutase (SODs) [101,102,103,104].

Interestingly, Lu and colleagues proved that curcumin administration could ameliorate GDM by increasing AMP-activated protein kinase (AMPK) activation in the livers of GDM mice and ameliorating oxidative stress by increasing Catalase (CAT), thiobarbituric acid reactive substance (TBARS), glutathione (GSH) and superoxide dismutase (SOD) levels [105].

Moreover, Wu and colleagues showed that curcumin administration reduced the levels of the lipid peroxidation marker by blocking ER stress, suggesting that curcumin supplementation may reduce the negative effects of diabetes on the embryo [106].

In addition, increased levels of pro-inflammatory cytokines such as TNF-a, IL-8 and IL-6 have been reported in pregnancy complicated by GDM, suggesting that proinflammatory cytokines could be involved in the development of insulin resistance associated to GDM [107,108]. Interestingly, it has been shown that curcumin was able to inhibit high glucose-induced inflammatory condition by interfering with the ROS/PI3K/AKT/mTOR signaling pathway and reducing secretion levels of TNF-α, IL-6 and IL-1β [45].

Another characteristic effect of GDM is the endothelial dysfunction due to the impairment of the uterine arteries that control the blood flow to the placenta. In fact, in GDM pregnancies there is an increased expression of markers of endothelial cell dysfunction such as soluble intercellular adhesion molecule (sICAM-1) and the soluble vascular cell adhesion protein 1 (sVCAM-1) [109,110], probably due to the inflammatory status characterizing GDM pregnancies [111].

Interestingly, many in vivo and in vitro studies proved the beneficial effects of curcumin in improving endothelial dysfunction due to diabetes [112], cadmium exposure [113], stroke [114] and other injuries [115,116].

Although insulin is recommended to treat hyperglycaemia in GDM pregnancies, there are also some side effects such as weight gain and hypoglycaemia [117]. For this reason, all these studies mentioned above show that curcumin administration may ameliorate many of the pregnancy dysfunctions due to GMD, suggesting curcumin as an alternative treatment for GDM.

## 6. Curcumin in Preeclampsia (PE)

Preeclampsia (PE) is a persistent hypertensive gestational disease which appears from the second trimester of pregnancy and is clinically characterized by de novo maternal hypertension (>140/90 mm Hg systolic/diastolic blood pressure) and proteinuria (>300 mg/24 h). In severe cases, the mother may develop comorbidities such as eclampsia, hepatic alterations (HELLP syndrome), edema and disseminated vascular coagulation (DIC). The main complications for the fetus due to PE are FGR, prematurity, and fetal death [118,119].

Over the last decade, there has been much progress in understanding the pathophysiology of this disease. In particular, it has been understood that PE originates from an impaired invasion of the extravillous trophoblasts (EVT) into the maternal uterine wall compromising the remodeling of the spiral uterine arteries and leading to hypoxic pregnancy condition [27]. This condition is a favorable environment for developing oxidative stress with consequent production of pro-inflammatory cytokines [120]. Normally, all cells are subject to oxidative stress but during pregnancy this is further accentuated due to the high oxygen and metabolic demands of the mother and fetus [121]. This leads to an increase in ROS production which can damage placental cells. Although cells developed antioxidant enzymes such as glutathione peroxidase (GSH-Px), glutathione (GSH), catalase (CAT) and superoxide dismutase (SOD) to scavenge ROS and prevent cellular damage, their activity is reduced during PE due to the low oxygen tension characterizing this pathology [122]. Thus, oxidative stress is a crucial process in the pathophysiology of PE, playing a key role in many placental disfunctions associated to this pathology. For this reason, antioxidant compounds such as curcumin might protect placental trophoblast cells during pregnancy, reducing oxidative stress and improving pregnancy outcome.

Recent studies have shown many beneficial effects on the use of curcumin as a cytoprotective compound against oxidizing agents. In particular, Qi and colleagues proved an anti-apoptotic action of curcumin (5 μM curcumin for 24 h) in human trophoblast HTR8/SVneo cells treated with H_2_O_2_, a well-established stimulator of oxidative stress in vitro [123], by increasing the Bcl-2/Bax ratio and decreasing the expression of cleaved-caspase 3 protein. Moreover, curcumin pre-treatment increased the expression of antioxidative enzymes NADP(H) quinine oxidoreductase 1 (NQO1) and heme oxygenase-1 (HO-1) by activating the NFE2-related factor-2 (Nrf2) signaling pathway [124].

Another important process that can dramatically influence the outcome of pregnancy is the placental angiogenesis, defined as the formation of new blood vessels from the existing vasculature network [125]. In fact, when this process is impaired there is an aberrant vascularization that leads to an imbalance between pro-angiogenic and anti-angiogenic factors. In PE the angiogenesis is impaired; in fact, in placental tissues of pregnancies complicated by PE there is an overexpression of soluble endoglin (sEng) and soluble fms-like tyrosine kinase-1 (sFlt1), two important anti-angiogenic proteins, while the expression of vascular endothelial growth factor (VEGF) and placental growth factor (PlGF), two important pro-angiogenic factors, is reduced [126,127].

Interestingly, Basak and colleagues showed that low doses (1–10 µM) of curcumin stimulated growth, proliferation, and viability in HTR8/SVneo cells. Moreover, they proved that curcumin was able to increase tube formation, and protein expression of proangiogenic factors such as VEGF receptor-2 (VEGFR2) and fatty acid-binding protein-4 (FABP4). In addition, curcumin was able to increase the expression of HLA-G, an important immuno-modulator and pro-angiogenic protein [128,129], promoting the immune environment to favor the invasive trophoblast cells [47].

The exposure of pregnant mice to LPS, one of the most powerful bacterial virulence factors with proinflammatory properties [130], has a double effect in in vivo studies as it allows the study of the role of infections in pregnancy but, due to its effects on the inflammatory status of the animal, it allows the mimicking of the conditions that are also found in pregnancy complications such as preeclampsia, FGR and pregnancy loss. In fact, exposure of pregnant rodents to LPS leads to increased inflammatory levels in the placenta, a condition usually found in these pathologies [131,132]. In particular, in pregnant mice exposed to LPS there is an increased systolic blood pressure and proteinuria in addition to increased placental IL-6, TNF-α and IL-1β expressions. Moreover, LPS exposure inhibits the activation of the PI3K/Akt signaling, an important pathway involved in cell survival, proliferation and an anti-inflammation modulator [133], by decreasing pAKT expression in mice placentas. Interestingly, curcumin administration was able to decrease systolic blood pressure, proteinuria and TNF-α, IL-1β, and IL-6 expressions in LPS-treated mice. In addition, curcumin administration increased the number of live pups, fetal weight, and placental weight probably due to increased pAKT levels in pregnant mice, suggesting that the anti-inflammatory effects of curcumin in LPS-treated mice could be due to the upregulation of phosphorylated Akt [134].

The beneficial effects of curcumin in LPS-induced preeclampsia-like phenotype was also proven in rat models. In fact, Gong and colleagues showed that curcumin was able to reduce blood pressure and proteinuria in LPS-curcumin-treated rats. Moreover, they also showed improved trophoblast invasion and spiral artery remodeling in curcumin-LPS-treated rats, reversing LPS-induced shallow placental implantation. In addition, they found that curcumin administration could decrease IL-6, MCP-1 protein expressions by inhibiting the TLR4/NF-κB signaling [135], a key pathway involved in pro-inflammatory cytokines production in preeclampsia [136] and a well-known target of curcumin [137].

## 7. Curcumin in Fetal Growth Restriction (FGR)

Fetal growth restriction is characterized by low birth weight of fetus during pregnancy and it has been shown to be associated to placental disfunction and increased placental reactive oxygen species production [138]. Thus, reducing oxidative stress in FGR pregnancy could alleviate placental oxidative damage found in FGR pregnancies. It has been found that in FGR mice, induced by low protein diet (8% proteins), serum progesterone levels, placental GSH-Px activity, MDA levels and antioxidant gene expression (Nrf2, HO-1, GCLC, NQO1, SOD1, SOD2 and CAT) were found to be decreased. Interestingly, curcumin addition to the mice’s diet significantly increased mRNA expression of Nrf2 and HO-1 in placental tissues and fetal growth, restoring redox balance by upregulating the expression of the antioxidant genes listed above [139]. This study showed that curcumin could exert antioxidant effect during pregnancy by activating the Nrf2/HO-1 pathway, which plays a key role in redox balance [140].

Similar results were obtained using FGR pigs induced by low protein diet integrated with curcumin [30,141,142]. In this study curcumin was able to increase piglets’ growth, to decrease hepatic lipid levels and insulin resistance. Moreover, curcumin supplementation ameliorates inflammation and oxidative damage, induced by FGR, increasing the expression of the NF-κB, JAK/STAT and activating the Nrf2/antioxidant response element (ARE) pathway in the liver.

Others reported that FGR-induced rats showed higher serum levels of glucose and increased HOmeostasis Model Assessment for Insulin Resistance index (HOMA-IR), TNF-α, IL-1β, IL-6, malondialdehyde (MDA), 8-hydroxy-2’-deoxyguanosine (8-OHDG) and higher activities of aspartate aminotransferase (AST) and alanine aminotransferase (ALT). In addition, FGR rats expressed high concentration of hepatic triacylglycerol (TAG), low activities of lipolysis enzymes and SOD. When their diet was supplemented with curcumin, concentrations of inflammatory cytokines, the activities of AST and ALT as well as the levels of MDA and 8-OHDG were decreased in the liver. Moreover, curcumin inhibited the phosphorylation levels of the NF-κB pathway and Janus kinase 2 (JAK2), increasing the expression levels of genes involved in the Nfe2l2/ARE pathway in the liver [143]. The same authors showed that curcumin supplementation decreased the concentrations of serum insulin, glucose and HOMA-IR in addition to reduced pyruvate, TAG, total cholesterol and non-esterified fatty acids (NEFA) in the liver by increasing the concentrations of glycogen and the activities of lipolysis enzymes in this organ. These results were due to the inhibition of phosphorylation of the insulin receptor substrate 1 (IRS-1), Akt, glycogen synthase kinase 3β (GSK-3 β) by curcumin [144].

These data showed that curcumin supplementation could prevent FGR-induced inflammation, oxidative damage and insulin resistance regulating insulin signaling pathways and inhibiting hepatic lipid accumulation by modulating NF-κB, JAK/STAT, Nrf2/HO-1 and Nrf2/ARE pathways.

## 8. Curcumin in Preterm Birth

Preterm birth, defined as babies born alive before 37 weeks of gestation, occurs in 11% of live births but it is one of the most important causes of poor pregnancy outcome leading to up to 75% of neonatal mortality [145]. It has been shown that inflammatory cytokines (IL-1β, IL-2, IL-6, IL-8 and TNF-α) [31], prostaglandins [146] and oxidative stress [32] play a key role in preterm birth by altering many mechanisms involved in pregnancy outcome.

Many in vitro and in vivo studies proved a beneficial role of curcumin in preventing preterm birth.

In fact, Guo and colleagues, by using C57BL/6 mice as model, showed that in pregnant mice exposed to LPS there is an increased expression of NF-κB, TNF-α and IL-8 in placental tissues. Moreover, IL-8 and MDA levels were also increased in the serum while live birth rate was decreased. Interestingly, they proved that in the LPS-curcumin-treated group, curcumin could prevent the activation of the NF-κB pathway, reducing the expression of TNF-α and IL-8 serum levels. Moreover, curcumin could relieve the damage of lipid peroxide induced by LPS increasing SOD expression and reducing MDA production leading to an increased live birth rate [147]. Lim and colleagues obtained similar results by in vitro study using placental explants, myometrial and primary amnion cells. They found that curcumin was able to reduce oxidative stress and NF-κB DNA-binding activity in all in vitro LPS-treated groups. Moreover, they found that curcumin significantly reduced the release of pro-inflammatory cytokines IL-6 and IL-8, decreased placental matrix metalloproteinase (MMP)-9, cyclooxygenase (COX-2) expression and inhibited the release of prostaglandins PGE2 and PGF2α in placenta explants and primary amnion cells previously stimulated by LPS and IL-1β. Differently, curcumin was not able to block IL-6 and IL-8 release in myometrial cells [148].

During normal pregnancy, the uterine stromal cells undergo numerous morphological and biological changes proliferating and differentiating to form the decidua. This process is tightly regulated during pregnancy and if it is altered it can compromise pregnancy outcome [20]. Uterine decidual cells are the major source of IL-6, a pro-inflammatory cytokine, which in pregnancy plays different roles in preterm birth [149]. It has been shown that IL-6 is increased in the decidual cell lines Huf and UIII when stimulated with IL-1β, leading to increased p50 and p65 expression, two subunits of NF-κB involved in NF-κB signaling. Moreover, IL-1β exposure increased STAT3 and IKK phosphorylation, two key players involved in inflammation induction [150]. Interestingly, curcumin was able to reduce IL-6 expression in IL-1β-treated decidual cells decreasing STAT3 and IKK phosphorylation. Moreover, curcumin dramatically inhibited both p50 and p65 protein expressions preventing their nuclear localization leading to NF-kβ signaling inhibition [150].

Taken together, in vitro and in vivo studies showed that curcumin could be a useful compound in preventing premature birth. In particular, it can counteract inflammation by inhibiting the expression of STAT3, NF-kβ transcription factors, reducing the expression of TNF-α, IL-8, IL-6, COX-2, PGE2, and PGF2a. Curcumin beneficial role in pregnancy is also due to its antioxidant capacity reducing the production of MDA and increasing SOD expression. Moreover, it can inhibit MMP expression reducing the risk of premature rupture of membrane, a key cause of preterm birth.

## 9. Conclusions and Further Research

The placenta is a complex organ, with multifaceted functions, essential for the normal development of humans and mammals in general. Its development is tightly regulated during pregnancy and changes in microenvironment may impair its normal development leading to the onset of a wide variety of pathological conditions with harmful impacts on fetal and maternal health. For these reasons, the use of any natural or synthetic compound during pregnancy requires particular attention as it could alter pregnancy processes still largely unknown.

It is known that excessive inflammation and oxidative stress play a key role in pregnancy pathological conditions such as PE, FGR, GDM and preterm birth impairing pregnancy outcome. It follows that the administration of anti-inflammatory and antioxidant compounds could be very important to prevent or ameliorate pregnancy complications. Non-Steroidal Anti-Inflammatory Drugs (NSAID) and glucocorticoids are excellent anti-inflammatory compounds, but they should not be used during pregnancy due to their side effects on the mother and fetus [151,152,153]. For this reason, it is always a challenge to find useful and safe products to treat these complications of pregnancy.

Although curcumin is generally considered safe in humans, there are still conflicting results on the use of curcumin in early pregnancy. In particular, curcumin showed harmful effects in oocyte maturation, fertilization and development of the blastocyst in animal studies.

In vitro and in vivo studies showed that curcumin positively modulates the main pathophysiological mechanisms involved in the most common complications related to pregnancy, including gestational diabetes mellitus, preeclampsia, FGR and preterm delivery (Table 1).

Moreover, curcumin showed protective effects against viral and bacterial infection in addition to protective action to damages induced by the exposure to natural and chemical toxic compounds during pregnancy.

In this review we reported the role of curcumin as a potent natural compound with anti-inflammatory and anti-oxidant properties. In particular, we highlighted the role of curcumin in modulating the expression of transcription factors as NF-κB, PPAR-γ and Akt, regulating signaling pathways including JAK/STAT, TLR4/NF-κB, Nrf2/HO-1 and Nfe2l2/ARE. Moreover, curcumin showed important functions in reducing production and secretion of pro-inflammatory cytokines such as TNF-α, IL-1β, IL-8 and IL-6. Curcumin was also able to modulate important chemokines (e.g MCP-1), prostaglandins (PGE2 and PGF2α), antioxidant enzymes (SOD, SOD, GSH, TBARS and CAT) and reduce levels of MDA and 8-OHDG.

To date, there are many clinical trials studying the efficacy of curcumin in many types of cancer that generally consider curcumin as well tolerated and efficient adjuvant therapy ameliorating the response to chemotherapy and radiotherapy reducing the side effects of these therapies [154,155,156,157].

Clinical effects of curcumin have also been shown in other pathologies. In fact, curcumin improved the severity of patients affected by non-alcoholic fatty liver diseases (NAFLD) disease decreasing the serum concentrations of inflammatory cytokines and chemokines such as TNF-α and MCP-1 [7]. Moreover, curcumin intake in women with polycystic ovarian syndrome (PCOS) significantly increased gene expression of PGC1α and activity of the Gpx enzyme reducing oxidative stress [158]. Another clinical trial showed that curcumin was able to improve the symptoms in patients affected by knee osteoarthritis [159]. Beneficial curcumin effects were also found in clinical trials focused on its role in body weight regulation, finding a role for curcumin in reducing BMI and increasing weight loss in addition to decreased serum levels of IL1β, IL-4 and VEGF [160,161]. Moreover, curcumin administration in two clinical trials showed that curcumin could decrease glucose levels in patients with type 2 diabetes mellitus [162,163].

However, to date there are no clinical studies regarding the role of curcumin in PE, FGR, GDM, preterm birth and viral or bacterial infections during pregnancy.

**Figure 2:**
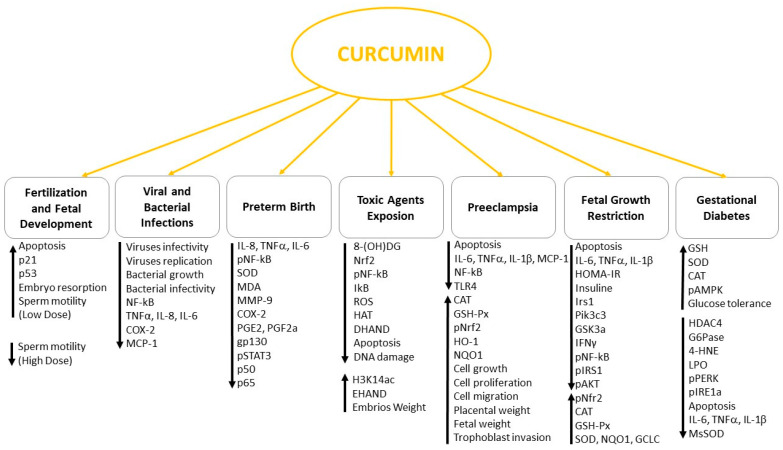
Curcumin effects on pregnancy disorders.

In conclusion, the use of curcumin in human diet is generally considered safe although more phytochemical studies of this natural product are needed to further evaluate its role in gametes maturation, fertilization and blastocyst development. Due to its multifaced role in regulating different signaling (Figure 2) and the encouraging data obtained in animal models and in vitro studies, curcumin intake during pregnancy could be beneficial in almost all the pregnancy complications mentioned in this review.

## Figures and Tables

**Table 1:** In vitro and in vivo actions of curcumin.

Curcumin Concentrations	Experimental Models	Outcomes	References
**Fertilization and Fetal Development**			
24 μM for 24 h	Mouse blastocyst	Increased ROS, apoptosis and embryo resorption; decreased fetal weight	[51]
24 μM for 24 h	Mouse blastocyst	Increased apoptosis at blastocyst stage or early egg stage and trophoblastic giant cell	[52]
20 μM for 24 h	Mouse blastocyst	Increased apoptosis, p21 and p53; decreased oocyte maturation and fertilization	[53]
1500, 3000 and 10,000 ppm for 70 days	Rat	No gross or microscopic changes in organs: no reproductive parameters changes; small reduction in pre-weaning body weight gain of the F2 pups at 10.000 ppm dose level.	[54]
0.015% of diet	Mouse and Rat	No effects on chromosomes, pregnancy rate, number of live and dead embryos	[55]
31.25–500 µM	Human and mouse spermatozoa	Decreased sperm motility starting from 62.5 µM but decreased capacitation/acrosome reaction at all concentrations tested	[57]
1 nM, 100 nM, 1 mM, 1 M	Human spermatozoa	Increased spermatozoa motility, reduced ROS formation and MDA production at 100 nM; decreased total and progressive motility at 1 mM and 1 M	[56]
**Protection against Cytotoxic and Teratogenic Agents**			
10 mg/Kg Curcumin ± 60 mg/Kg Retinoic Acid (RA) for 10 days	Mouse	Increased crown rump (CR) length and embryos weight; decreased cell number and sinusoid diameter in the embryonic liver tissue	[44]
250 mg/kg Curcumin (for 1 h) ± 1 mg/kg Aroclor1254 for 28 days	Rat	Decreased 8-(OH)DG, 5-methycytosine and 5-hydroxymethycytosine levels; decreased karyopyknotic nuclei and shrunken or swollen cytoplasm in the migrating neurons	[68]
16 g/Kg Curcumin ± 3 g/L Lead (Pb) for 82 days	Rat	Improved sensory and motor functions in neonatal rats	[73]
75 mg/kg/d Curcumin ± 6 g/kg/d alcohol for 8 days	Mouse	Decreased HAT activity, DHAND; increased H3K14ac, EHAND; inhibited H3K14ac connection with DHAND and EHAND	[75]
25 μM Curcumin ± 200 mM alcohol for 24 h	Cardiac progenitor cells	Decreased H3K9 acetylation and apoptosis reducing cleaved caspase-3 and cleaved caspase-8; increased bcl-2	[76]
100 mg/kg Curcumin ± 85 ppm Arsenic (As) for 10 days	Mouse	Increased number of EpASCs; decreased chromosomal aberrations; decreased AS accumulation in liver, skin, hair and kidney; decreased Nrf2, NFkB and IkB	[79]
500 nmol/kg Curcumin ± 30 mg/kg Celecoxib for 4 days	Mouse	Increased neurogenesis upregulating GSK-3B and β−Catenin	[84]
150 or 300 ppm Curcumin ± 10 ppm of HgCl_2_ for 35 days	Mouse	Increased body weight, anticipated hairgrowth and eye opening; increased memory, learning ability, and levels of dopamine, serotonin and acetylcholinesterase in forebrain of pups	[87]
5, 10 and 20 μM (for 1 h) Curcumin ± 200 μM Methylglyoxal (MG) for 3 h	Mouse embryonic stem cells and blastocyst	Decreased DNA fragmentation, caspase-3 activation, cleavage of PARP, JNK activation and apoptosis; decreased ROS production	[46]
**Viral and Bacterial Infections**			
5 μM for 2 h	HeLa, BHK-21, Vero-E6 cells; CHIKV, VSV, ZIKV viruses	Reduced infectivity of ZIKV and CHIKV viruses by blocking the binding of viruses to cell surface	[88]
0.2, 0.4 and 0.8 µg/mL for 2 days	HELF cells; HCMV virus	Reduced infectivity of HCML by decreasing Hsp90 protein expression	[90]
20 μM for 2 h	HeLa and Vero cells; HSV-1 virus	Reduced HSV-1 infectivity and replication by decreasing ICP4 and ICP27 genes in a p300-independent way	[91]
50 μM for 12 h	HEK-293T, J1.1, TZM-bl cells; HIV-1 virus	Inhibited HIV activity by degrading unfolded Tat protein in a proteasomal dependent way	[92]
0.25 μM for 30 min	Recombinant HIV-1 integrase	Inhibited HIV-1 activity by blocking integrase function	[93]
8 μg/mL for 30 min 200 mg/kg curcumin for 8 h	Mouse; J774 cells; *L. Monocytogenes*	Inhibited bacterial growth by interfering with the activity of listeriolysin O; protective effects against infection in mice	[94]
150 mg/kg for 14 days	*S. agalactiae;* silver catfish	Bactericidal action against *S. agalactiae;* prevented occurrence of clinical signs	[95]
100 μM for 1 h	HeLa cells; *Neisseria* *gonorrhoeae*	Interfered with bacterial binding to host cells in late infection; inhibited IkBa degradation and NF-kB activation; reduced TNFa, IL-8 and IL-6 secretion	[96]
40 mg/kg for 2 h	Mouse; LPS	Restored neuronal cell morphology in fetal brain; reduced production of IL-6, Il-1B, COX-2, sICAM-1, sE-selectin, CCL-2, MCP-1 and CINC-1	[97]
**Gestational Diabetes**			
100 mg/kg for 10 days	Mouse	Increased glucose tolerance by decreasing TBARS and increasing GSH, SOD and CAT; Enhanced AMPK activation and decreased HDAC4 and G6Pase expression	[105]
20 μM for 24 h	Mouse embryos	Reduced neuronal tube defects and levels of 4-HNE and LPO; blocked ER stress by inhibiting pPERK, pIRE1α, peIF2α, CHOP, BiP and XBP1; reduced cleaved caspase3 and 8	[106]
10 μM for 1 h	RPEC cells	Reduced glucose‑induced toxicity and TNF‑α, IL‑6 and IL‑1β levels inhibiting AKT and mTOR activation	[45]
7.5 mg/kg/day for 7 days	Endothelial progenitor cells (EPCs) from mouse	Restored tubule formation, and migration of EPCs; improved wound healing; reduced levels and activity of MnSOD.	[112]
**Preeclampsia (PE)**			
5 µM for 24 h	HTR8/SVneo cell line	Reduced apoptosis by increasing Bcl-2/Bax ratio and decreasing cleaved-caspase 3. Reduced oxidative stress by enhancing CAT and GSH-Px activity, and activating Nrf2. Increased HO-1, NQO1 expression.	[139]
5 µM for 24 h	HTR8/SVneo, JEG3 and HMEC-1 cell lines	Increased cell growth, migration, proliferation and viability by activating AKT; increased tube formation, VEGFR2 HLA-G and FABP4 expression; increased DNMT3A and HSD11B2 expression	[47]
100 µg/kg/d for 17 days	LPS-treated Mouse	Decreased systolic blood pressure, proteinuria, IL-6, IL-1B, TNFa, MCP-1 and MIP-1; increased live pups, fetal and placental weight; Decreased macrophages in placenta.	[134]
360 µg/kg for 14 days	LPS-treated Rat	Decreased systolic blood pressure and proteinuria; improved trophoblast invasion and spiral artery remodeling; decreased TLR4, NF-κB, IL-6 and MCP-1 expressions	[135]
**Fetal Growth Restriction (FGR)**			
400 mg/kg/d for 18 days	Mouse	Decreased placental apoptosis; increased blood sinusoids area, CAT and GSH-Px in placenta; increased Nef2, HO-1, SOD2, CAT, NQO1 and GSH-Px in fetal liver	[139]
400 mg/kg for 24 days	Pig	Decreased TNF-α, IL-1β and IL-6 serum levels; increased body weight; reduced insulin, glucose, and HOMA-IR levels by downregulating Irs1, Pik3c3, and Gsk3a	[30]
200 mg/kg for 90 days	Pig	Increased Nrf2, SOD1, GCLC, GCLM, NQO1; decreased levels of TNFα, IL-6, IFNγ and caspase3, bax, bcl2, hsp70 mRNA expression.	[141]
400 mg/kg for 24 days	Pig	Increased body-weight gain and Nrf2 and Hmox1 proteins in the liver of FGR piglets	[142]
400 mg/kg for 6 weeks	Rat	Decreased TNF-α, IL-1β, IL-6, AST and ALT in the serum; decreased pNF-κB, pJAK2 and increased mRNA expression of genes of the Nfe2l2/ARE pathway in the liver.	[143]
400 mg/kg for 6 weeks	Rat	Decreased insulin, glucose, HOMA-IR, pyruvate, TAG, total cholesterol and NEFA in the liver; decreased pIRS1, pAKT, pGSK-3, FASN and SREBP-1 in liver.	[144]
**Preterm Birth**			
100 mg/kg for 24 h	Mouse	Decreased NF-kBp65, TNFa and IL-8 in placental tissue; decreased serum levels of IL-8, SOD and MDA; increased live birth rate.	[147]
30 and 60 µM for 24 h	Placental explants, Primary amnion cells and Myometrial cells	Decreased IL-6, IL-8, MMP-9, COX-2, PGE2 and PGF2a; decreased 8-Isoprostane and NF-kB/DNA binding.	[148]
5, 10, 20, 30 and 40 µM for 1, 2 and 24 h	HuF and UIII cells	Decreased IL-6, gp130, pSTAT3, p50 and p65 expression	[150]
